# High driving pressure ventilation induces pulmonary hypertension in a rabbit model of acute lung injury

**DOI:** 10.1186/s40560-023-00689-w

**Published:** 2023-09-25

**Authors:** Yonghao Xu, Yu Zhang, Jie Zhang, Weibo Liang, Ya Wang, Zitao Zeng, Zhenting Liang, Zhaoyi Ling, Yubiao Chen, Xiumei Deng, Yongbo Huang, Xiaoqing Liu, Haibo Zhang, Yimin Li

**Affiliations:** 1 Department of Critical Care Medicine, The First Affiliated Hospital of Guangzhou Medical University, Guangzhou Institute of Respiratory and Health, Medical Center for Respiratory Medicine, State Key Laboratory of Respiratory Disease, Guangzhou, 510120 China; 2 The Keenan Research Centre for Biomedical Science, St Michael’s Hospital, Toronto, ON M5B1W8 Canada; 3 Department of Anesthesia, University of Toronto, Toronto, ON Canada; 4 Interdepartmental Division of Critical Care Medicine, University of Toronto, Toronto, ON Canada

**Keywords:** Acute respiratory distress syndrome, Pulmonary hypertension, Mechanical ventilation, Ferroptosis

## Abstract

**Background:**

Mechanical ventilation may cause pulmonary hypertension in patients with acute lung injury (ALI), but the underlying mechanism remains elucidated.

**Methods:**

ALI was induced in rabbits by a two-hit injury, i.e., hydrochloric acid aspiration followed by mechanical ventilation for 1 h. Rabbits were then ventilated with driving pressure of 10, 15, 20, or 25 cmH_2_O for 7 h. Clinicopathological parameters were measured at baseline and different timepoints of ventilation. RNA sequencing was conducted to identify the differentially expressed genes in high driving pressure ventilated lung tissue.

**Results:**

The two-hit injury induced ALI in rabbits was evidenced by dramatically decreased PaO_2_/FiO_2_ in the ALI group compared with that in the control group (144.5 ± 23.8 mmHg vs. 391.6 ± 26.6 mmHg, *P* < 0.001). High driving pressure ventilation (20 and 25 cmH_2_O) significantly elevated the parameters of acute pulmonary hypertension at different timepoints compared with low driving pressure (10 and 15 cmH_2_O), along with significant increases in lung wet/dry ratios, total protein contents in bronchoalveolar lavage fluid, and lung injury scores. The high driving pressure groups showed more pronounced histopathological abnormalities in the lung compared with the low driving pressure groups, accompanied by significant increases in the cross-sectional areas of myocytes, right ventricular weight/body weight value, and Fulton’s index. Furthermore, the expression of the genes related to ferroptosis induction was generally upregulated in high driving pressure groups compared with those in low driving pressure groups.

**Conclusions:**

A rabbit model of ventilation-induced pulmonary hypertension in ALI was successfully established. Our results open a new research direction investigating the exact role of ferroptosis in ventilation-induced pulmonary hypertension in ALI.

## Background

Acute respiratory distress syndrome (ARDS) is a clinical syndrome characterized by severe hypoxemia and respiratory distress, with a high mortality of approximately 30–40%. The main treatment for ARDS is lung-protective ventilation with prone positioning and neuromuscular blockade [1]. Pulmonary hypertension along with right ventricular dysfunction or failure is significantly correlated with poor outcomes of ARDS [2] when compared with hypoxemia [3, 4]. A prospective observational study including 752 patients with moderate and severe ARDS has reported a 22% incidence of acute cor pulmonale and a poorer outcome in severe acute cor pulmonale [5]. The clinical expression of pulmonary hypertension includes increased pulmonary arterial pressure and the development of right ventricular dilatation and dysfunction [6]. In ARDS management, mechanical ventilation-related pulmonary hypertension may cause right ventricular failure due to preload insufficiency or excessive afterload [7]. Although mechanical ventilation may open the lung and improve arterial oxygen saturation, it is also associated with the risk of pulmonary vascular injury and right ventricular injury [8]. Thus, cardio-pulmonary protective ventilation is required for ARDS patients. However, the underlying mechanism of ventilation-induced pulmonary hypertension (VIPH) in ARDS remains elusive and thus preclinical studies including animal models of VIPH may help examine mechanistic insights for one to reduce and prevent VIPH from happening in ARDS.

This study aimed to establish an animal model of VIPH following established ALI mimicking clinical ARDS and examine gene profiles in lung tissue to identify differentially expressed genes (DEGs) that may contribute to its occurrence. Our study provides an ideal animal model for the research of protective ventilation in ALI and suggests that ferroptosis-related genes might play important roles in the development of VIPH that may serve as future therapeutic target for VIPH in ARDS.

## Methods

### Animals

This study was approved by the Laboratory Animal Ethics Committee of Affiliated First Hospital of Guangzhou Medical University Hospital (Approval No. HTSW211023). Animal experiments were performed following the Guide for the Care and Use of Laboratory Animals [9]. Male adult New Zealand white rabbits weighing 3.32 ± 0.16 kg were purchased from Guangdong Medical Laboratory Animal Center (Guangdong, China) and housed following the animal protection laws of China with free access to food and water.

### Mechanical ventilation and baseline characterization

**Fig. 1:**
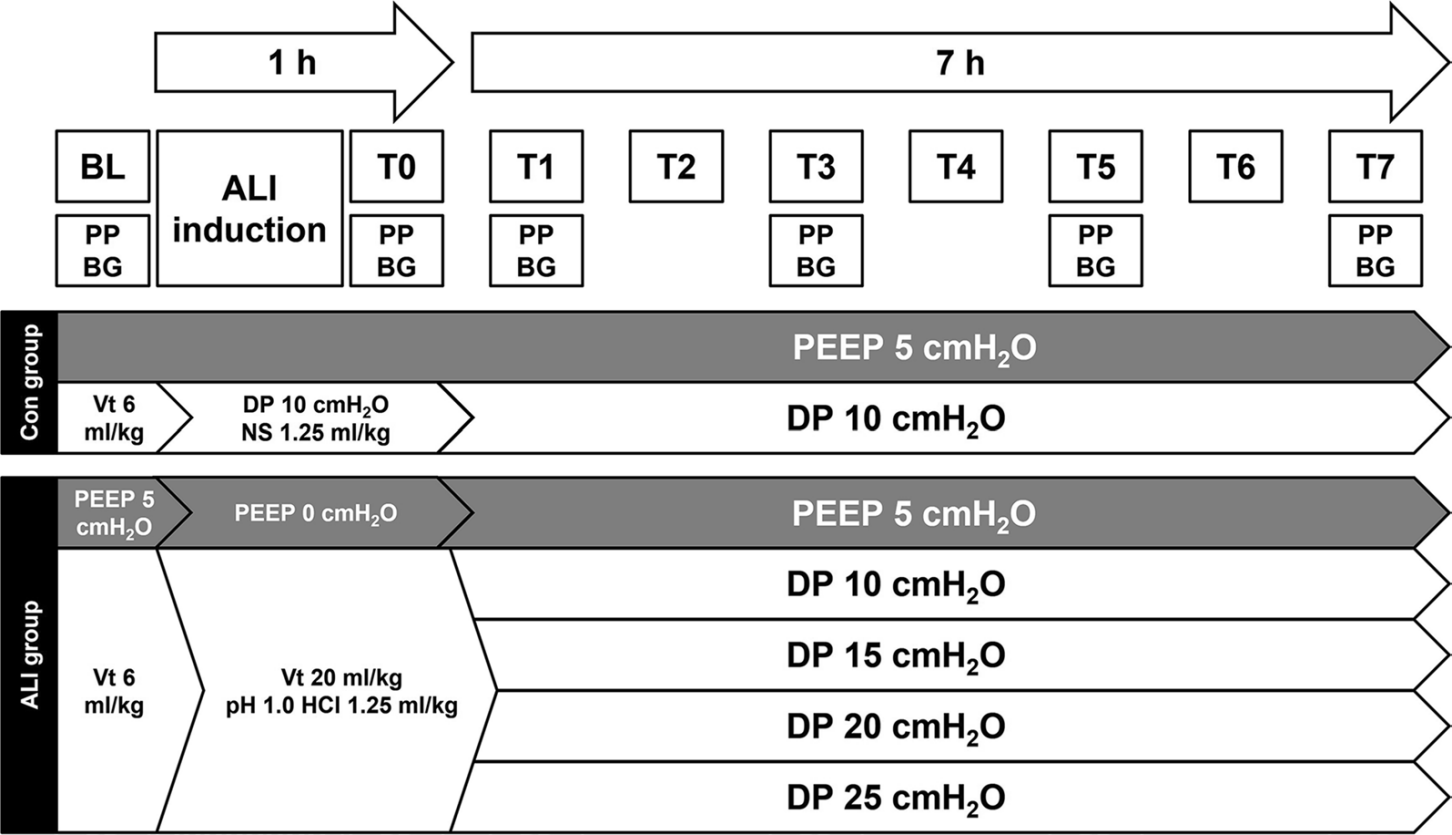
Pulmonary artery cannulation. **A** Wire was inserted into a catheter, and the end of the catheter was bent. **B** Catheter after thermal modification; **C** guide wire was inserted into the catheter to make the end of the catheter become 90°; **D** guide wire was pulled out, and the end of the catheter was bent back to 180°; **E** catheter was placed in the right ventricle; **F** monitor depicted a right ventricular waveform; **G** catheter was laid into the pulmonary artery; **H** typical pulmonary artery pressure waveform

The workflow of the animal study is shown in Fig. 1. All rabbits were anesthetized with urethane (1.0 g/kg) via marginal ear vein injection, followed by a continuous intravenous infusion of urethane (250 mg/kg/h) via a 24G catheter laid into the marginal ear vein to maintain the anesthesia. Continuous infusions of pancuronium bromide (0.2 mg/kg/h), NaHCO_3_ (0.5 mmol/kg/h), and 0.9% NaCl (10 mL/kg/h) were administered for neuromuscular blockade, acid–base balance, and body fluid balance, respectively. Then, a tracheostomy was carried out after infiltrating the surgical site with lidocaine, and an uncuffed tube with an internal diameter of 4.0 mm was inserted into the trachea. Mechanical ventilation was initiated at a tidal volume of 6 mL/kg and a respiratory rate of approximately 80 breaths/min to achieve normocapnia using the volume-controlled mode of a pediatric ventilator (Servo-i, Maquet Critical Care, Solna, Sweden) with a fraction of inspired oxygen (FiO_2_) of 0.4 and positive end-expiratory pressure (PEEP) of 5 cmH_2_O. A 3 French pulse index continuous cardiac output catheter (Pulsion Medical System, Munich, Germany) was placed in the right femoral artery for invasive blood pressure monitoring, intermittent blood withdrawal for arterial blood gas analysis, and cardiac output measurement. Another 3 French catheter was inserted into the right jugular vein to measure central venous pressure and thermodilution cardiac output. Vital parameters stabilized at approximately 5 min after the surgery, and the baseline parameters were measured. Airway pressure, arterial pressure, pulmonary artery pressure, central venous pressure, and cardiac output were continuously recorded. Mean arterial pressure (MAP) and mean pulmonary artery pressure (mPAP) were calculated.

### Pulmonary artery cannulation

**Fig. 2:**
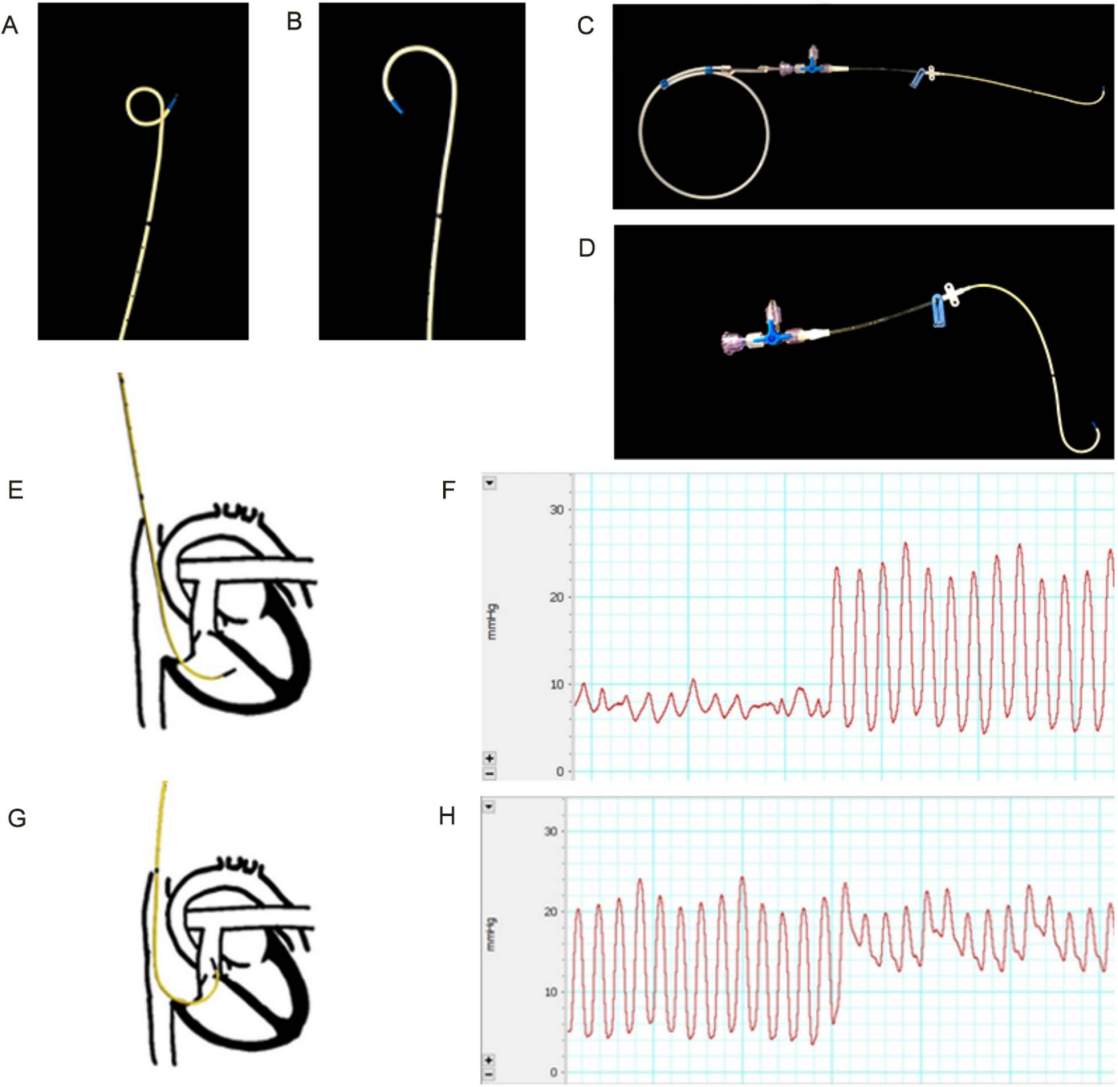
Schematic representation of the study. A total of 25 rabbits were randomly assigned to control group (*n* = 5) and acute lung injury (ALI) group (*n* = 20). After measuring the baseline physiological parameters, rabbits in the ALI group were treated with hydrochloric acid aspiration (1.25 mL/kg) followed by 1-h 10 cmH_2_O mechanical ventilation to induce ALI. Physical parameters were recorded at T0. Rats with ALI were randomized into four groups (*n* = 5/group) and ventilated with a driving pressure of 10, 15, 20, or 25 cmH_2_O with a positive end-expiratory pressure of 5 cmH_2_O for 7 h. The control group was ventilated with a driving pressure of 10 cmH_2_O for 7 h. Physical parameters were collected every 2 h at T1, T3, T5, and T7. BL: baseline; PP: physiological parameters; BG: blood gas; DP: driving pressure; PEEP: positive end-expiratory pressure; NS: normal saline; HCl: hydrochloric acid; Con: control; ALI: acute lung injury

A 30 cm wire was inserted into a 16G single-lumen central venous catheter (Arrow, Teleflex Medical Europe, Westmeath, Ireland), and the end of the catheter was bent to 300° to form a circle with a diameter of 1.5 cm (Fig. 2A). After soaking in water at 80 °C for 10 min, the wire was immediately transferred into ice-cold water for 10 min. The end of the catheter was bent to about 180° (Fig. 2B). The guide wire was inserted into the catheter to make the end of the catheter become 90° (Fig. 2C). After removing the metal guide wire, the end of the catheter became 180° (Fig. 2D). To insert the catheter into the right ventricle (RV), the jugular vein was exposed and separated, with the distal end ligated and the proximal end clamped. The catheter with guide wire was inserted into the jugular vein and ligated to ensure that the blood does not leak and the catheter could move smoothly (Fig. 2E). Then, the image of the physiological monitor switched from a right atrium waveform to a right ventricular waveform (Fig. 2F). To insert the catheter into the pulmonary artery, the guide wire was pulled out, so that the catheter returned to its initial curvature. Then, the catheter was slowly inserted 1 to 2 cm into the pulmonary artery (Fig. 2G). When a typical pulmonary artery pressure waveform appeared (Fig. 2H), the catheter was pulled 1 to 2 mm out of the pulmonary artery and fixed.

#### ALI model

An ALI model was induced in rabbits by a two-hit injury, i.e., hydrochloric acid aspiration followed by mechanical ventilation for 1 h, as previously described [10]. Briefly, the rabbits were randomly divided into control group (*n* = 5) and ALI group (*n* = 20). The control group received a single dose of normal saline aspiration at 1.25 mL/kg. Each rabbit in the ALI group was administered a single dose of hydrochloric acid (pH 1.0) aspiration at 1.25 mL/kg, followed by an injurious ventilation (zero-end-expiratory pressure, tidal volume = 20 mL/kg, and FiO_2_ = 1.0) for 1 h. The control group was not given injurious ventilation. Blood gas measurements and vital parameters were recorded (T0). The respiratory rate was adjusted to keep the partial pressure of arterial carbon dioxide (PaCO_2_) at 35 to 45 mmHg. When the ratio of arterial oxygen partial pressure and fraction of inspired oxygen (PaO_2_/FiO_2_) reached approximately 100 to 200 mmHg, the animals were randomized into four groups (*n* = 5/group) and ventilated with a driving pressure of 10, 15, 20, or 25 cmH_2_O. The control group was ventilated with a driving pressure of 10 cmH_2_O. All groups were continuously ventilated for 7 h with PEEP of 5 cmH_2_O. Data were collected every 2 h (T1, T3, T5, and T7) (Fig. 2). In the cohort subjected to a 25 cmH_2_O ventilatory regimen, we observed that two out of the initial five animals succumbing during the ventilation procedure due to individual variations and possibly differences in lung injury severity. To uphold the integrity of the subsequent statistical analysis, it became imperative to address this unforeseen loss. Consequently, we introduced two supplementary animals of matching weight and gender into the same group. This strategic inclusion was undertaken to restore group composition equilibrium and ensure the robustness of our statistical evaluations. The surviving animals were euthanized with an overdose of intravenous Urethane (2 g/kg) under general anesthesia at 7 h after ventilation. Lung tissue samples were immediately harvested.

### Blood gas analysis

PaO_2_, PaCO_2_, and HCO_3_^−^ were determined using a point-of-care blood gas analyzer (i-STAT, Abbott Laboratories, Chicago, IL, US).

### Hematoxylin and eosin (H&E) staining

The left lung was perfused with 4% paraformaldehyde at a hydrostatic pressure of 20 cmH_2_O. Lung tissue sections (4 μm) were stained with H&E and blindly analyzed by two experienced pathologists following American Thoracic Society guidelines [11, 12]. Five parameters were scored in 20 high-power fields in each slide, including neutrophil infiltration in the alveolar space (A), neutrophil infiltration in the interstitial space (B), hyaline membranes (C), proteinaceous debris in the airspaces (D), and alveolar septal thickening (E). The lung injury score was calculated as [(20 × A) + (14 × B) + (7 × C) + (7 × D) + (2 × E)]/(the number of fields × 100). Heart ventricles were excised, fixed in 4% paraformaldehyde, and stained with H&E. The cross-sectional areas of the myocytes were determined using an automated image analysis system in Image-J 2.3.0. Myocytes from randomly selected five fields of each sample were counted.

### Lung wet/dry weight ratio

The wet/dry weight ratio was calculated to assess lung tissue edema. Briefly, the right middle lobe of the right lung was harvested and weighed (wet weight). After heating at 80 °C for 48 h, the lung sample was weighed again (dry weight). The wet/dry weight ratio was calculated.

### Bronchoalveolar lavage fluid (BALF) analysis

BALF was obtained by perfusing the right upper lobe with 5 mL normal saline three times, followed by centrifugation for 10 min at 1500 rpm and 4 °C to remove the cells. Protein concentration was measured using a BCA protein assay kit.

### Determination of right ventricular remodeling

The hearts excised from the rabbit were washed with phosphate buffered saline. The right ventricle (RV) and left ventricle plus interventricular septum (LV + S) were separated, and their dry weights were determined. Right ventricular remodeling was evaluated by calculating the Fulton index, i.e., RV/[LV + S].

### RNA sequencing

Total RNA was extracted from the lung using TRIzol reagent according to the manufacturer’s instructions (Magen, China). The quantity and quality of RNA samples were examined using a Nanodrop ND-2000 system (Thermo Scientific, USA) and an Agilent Bioanalyzer 4150 system (Agilent Technologies, CA, USA). Paired-end libraries were prepared using an ABclonal mRNA-seq Lib Prep Kit (ABclonal, China) following the manufacturer’s instructions. The mRNA generated from 1 μg total RNA was purified using oligo magnetic beads, followed by fragmentation using divalent cations at elevated temperatures in ABclonal first strand synthesis reaction buffer. Then, the first-strand cDNAs were synthesized using random hexamer primers and reverse transcriptase, followed by second-strand cDNA synthesis. The double-stranded cDNAs were adapter-ligated for PCR amplification, followed by purification using an AMPure XP system. After quality control on an Agilent Bioanalyzer 4150 system, the library was sequenced on an Illumina Novaseq 6000 or MGISEQ-T7 instrument. The 150 bp paired-end reads were generated. The raw data were analyzed using an in-house pipeline from Shanghai Applied Protein Technology (China). The genes with adjusted *P* value < 0.05 and |log2 fold change|> 1 were identified as DEGs. Heatmap was generated using Z-scores.

### Statistical analysis

Data were expressed as the mean ± standard deviation and compared using one-way or two-way analysis of variance. All data were analyzed using SPSS 26.0 software (SPSS Inc., Chicago, IL, USA). Graphs were generated using GraphPad Prism 9.4.0 software (GraphPad, CA, USA). A *P* value < 0.05 was considered statistically significant.

## Results

### High driving pressure ventilation impairs lung function in rabbits with two-hit injury-induced ALI.

**Fig. 3:**
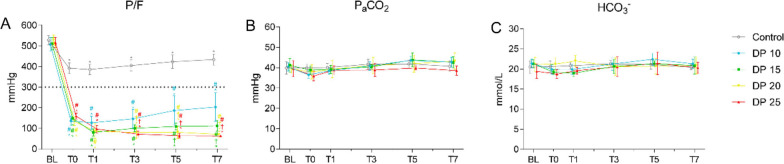
Blood gas parameters at different timepoints during ventilation. Blood gas parameters were measured at baseline, T0, T1, T3, T5, and T7. Data are presented as the means ± standard deviation (SD). *P < 0.05 vs. baseline; ^#^*P* < 0.05 vs. control; ^†^*P* < 0.05 vs. 10 cmH_2_O. *n* = 5. P/F: the ratio of arterial oxygen partial pressure and fraction of inspired oxygen; PaCO_2_: arterial partial carbon-dioxide pressure; HCO_3_^−^: bicarbonate; DP: driving pressure

To induce ALI in rabbits, we applied a two-hit injury of hydrochloric acid aspiration followed by injurious mechanical ventilation for 1 h. All experimental groups were able to complete the 7-h mechanical ventilation period except for the 25 cmH_2_O group. The data from the two deceased animals in the 25 cmH_2_O group at T0, including body weight, DP, MAP, CO, mPAP, PASP, PVRI and ELWI showed no statistically significant differences when compared to the baseline of other animals in the same group (data not shown). As shown in Fig. 3A, the PaO_2_/FiO_2_ value of the ALI group dramatically dropped from around 515.8 ± 27.3 mmHg at baseline to 144.5 ± 23.8 mmHg at T0, suggesting that ALI was successfully induced in rabbits. To investigate the association of driving pressure with lung injury in ALI, we ventilated ALI rabbits with low driving pressure (10 and 15 cmH_2_O) or high driving pressure (20 and 25 cmH_2_O) for 7 h. We found that mechanical ventilation reduced the PaO_2_/FiO_2_ value of ALI rabbits in a driving pressure-dependent manner starting T3. No significance was observed between 20 cmH_2_O and 25 cmH_2_O groups at any timepoint. Of note, the PaO_2_/FiO_2_ value of the 10 cmH_2_O group tended to increase from T1 to T7, whereas the PaO_2_/FiO_2_ values of 15, 20, and 25 cmH_2_O groups remained stable and substantially lower than those of the 10 cmH_2_O group from T1 to T7. PaCO_2_ and HCO_3_^−^ showed no difference among different groups at any timepoint (Fig. 3B, and C). These results suggest that 10 cmH_2_O ventilation might improve lung function and that ventilation with driving pressure higher than 10 cmH_2_O may result in persistent lung injury.

### High driving pressure ventilation induces pulmonary hypertension in rabbits with ALI

**Fig. 4:**
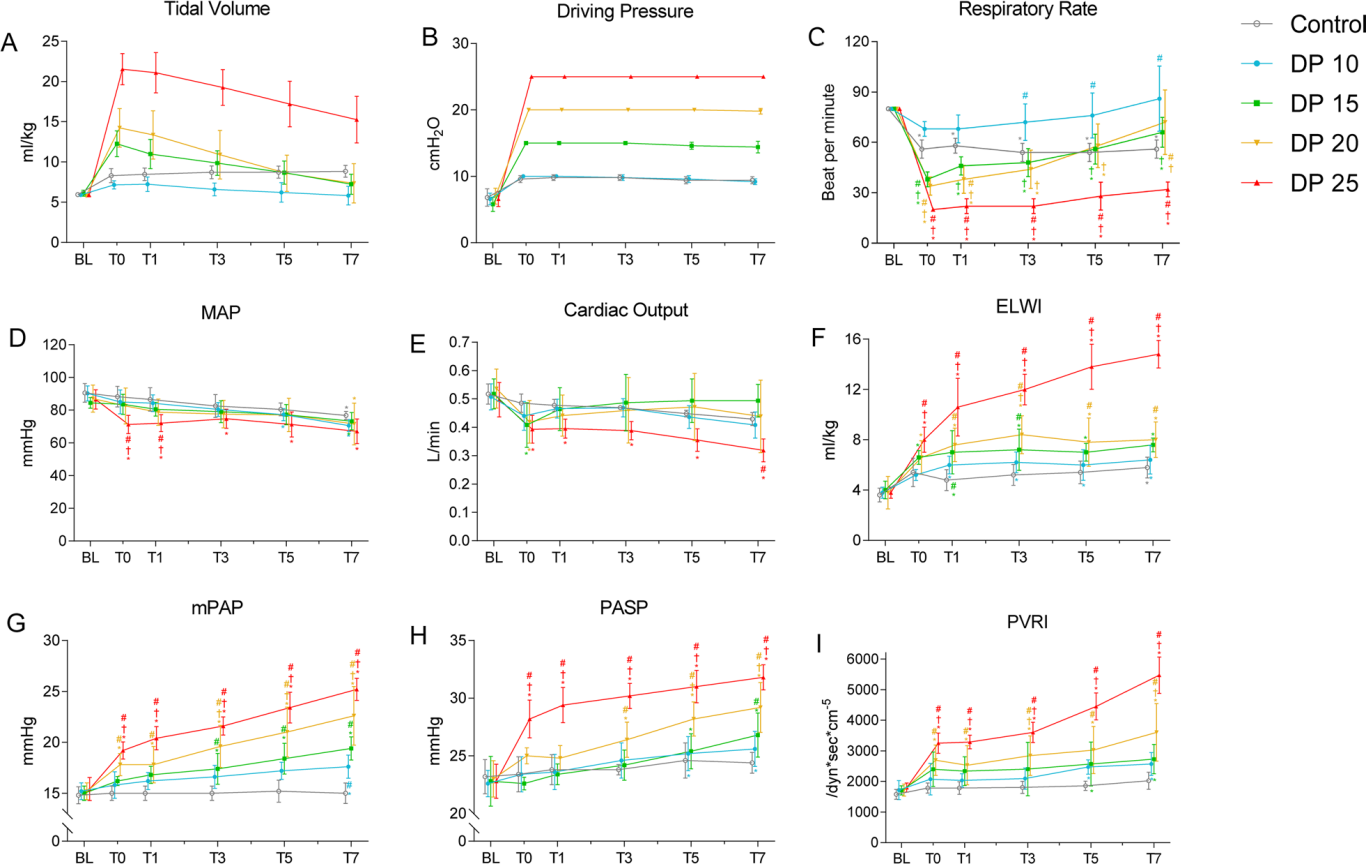
Parameters of hemodynamics and respiratory mechanics at different timepoints during ventilation. Parameters of hemodynamics and respiratory mechanics were measured at baseline, T0, T1, T3, T5, and T7. Data are presented as the means ± SD.*P < 0.05 vs. baseline; ^#^*P* < 0.05 vs. control; ^†^*P* < 0.05 vs. 10 cmH_2_O. *n* = 5. MAP: mean arterial pressure; mPAP: mean pulmonary arterial pressure; PASP: pulmonary arterial systolic pressure; PVRI: pulmonary vascular resistance index; ELWI: extravascular lung water index

To investigate the association of driving pressure with VIPH in ALI, we measured hemodynamic parameters in rabbits. We found that with the driving pressure remaining constant, the tidal volumes of the 15, 20, and 25 cmH_2_O groups were remarkably and pressure-dependently increased at T0 compared with those at baseline and gradually decreased thereafter (Fig. 4A, and B). Conversely, the respiratory rates of these groups showed a notable and pressure-dependent decrease at T0 in comparison with the baseline, but they gradually increased thereafter (Fig. 4C). The results suggest that high driving pressure ventilation impairs lung compliance. The mean arterial pressure and cardiac output of the 25 cmH_2_O group were significantly lower than those of the control group across T0 to T7, whereas the parameters of other ventilation groups remained stable and comparable to those of the control groups across T1 to T7 (Fig. 4D, and E). In contrast, the mean pulmonary arterial pressure, pulmonary arterial systolic pressure, pulmonary vascular resistance index, and extravascular lung water index were generally increased in all ventilated ALI rabbits compared with those in control rabbits in a pressure- and time-dependent manner. Specifically, 20 cmH_2_O and 25 cmH_2_O ventilation resulted in more pronounced increases in these parameters in ALI rabbits than low driving pressure (Fig. 4F–I). Altogether, these data suggest that high driving pressure ventilation causes acute pulmonary hypertension in ALI.

### High driving pressure ventilation aggravates pulmonary vascular injury in rabbits with ALI

**Fig. 5:**
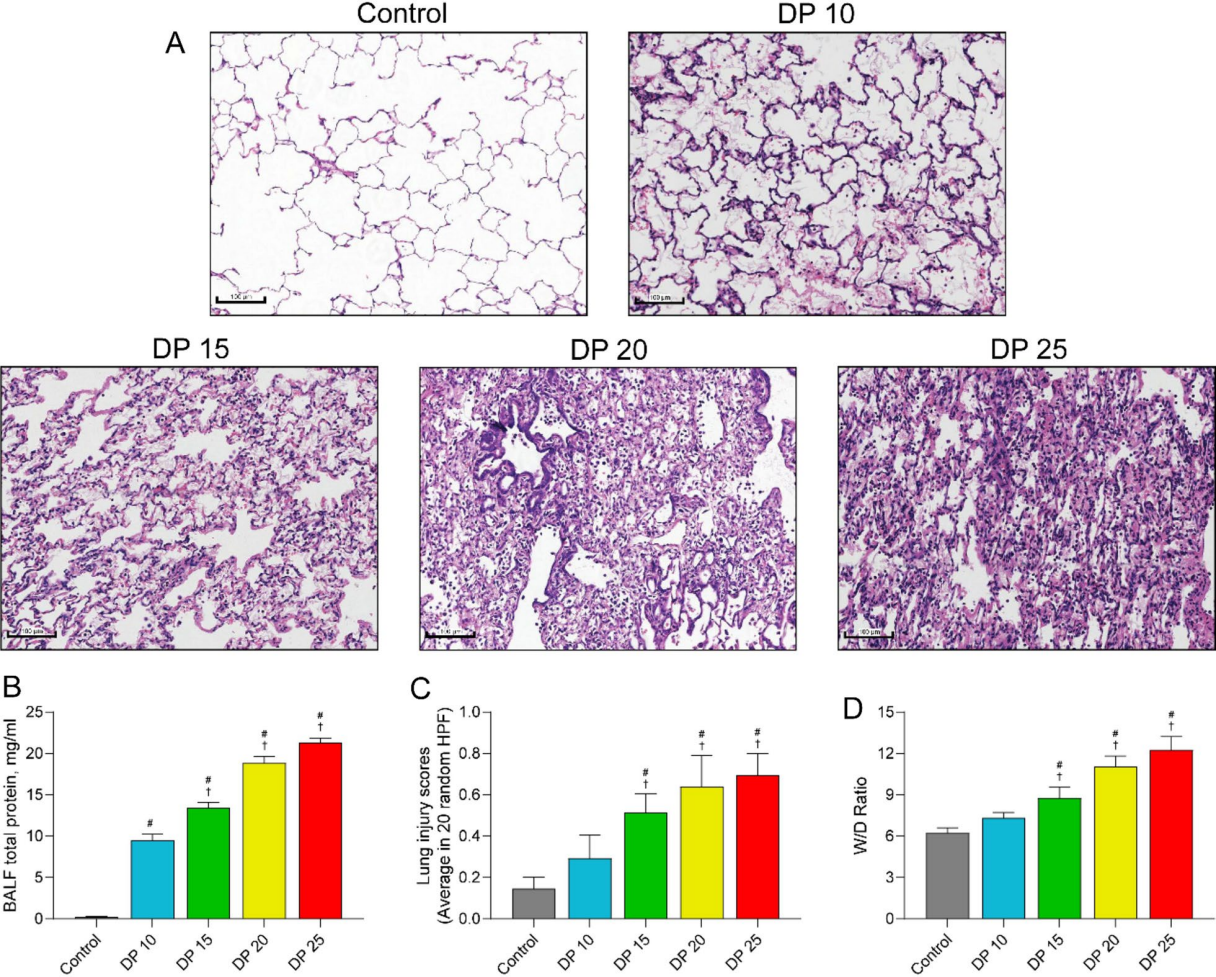
High driving pressure aggravated ALI. **A** Rabbit lung tissue samples were collected at 7 h after ventilation and stained with hematoxylin and eosin. Images were acquired at magnification ×200. **B** Bronchoalveolar lavage fluid was obtained, and protein concentration was measured. **C** Lung injury scores were calculated in 20 high-power fields per animal. **D** Lung tissue was immediately weighed after collection and reweighted after drying at 80 °C for 48 h. The wet/dry ratio was calculated. Data are presented as the means ± SD. ^#^*P* < 0.05 vs. control; ^†^*P* < 0.05 vs. 10 cmH_2_O; *n* = 5. W/D: wet-to-dry weight ratio; DP: driving pressure; BALF: bronchoalveolar lavage fluid

**Fig. 6:**
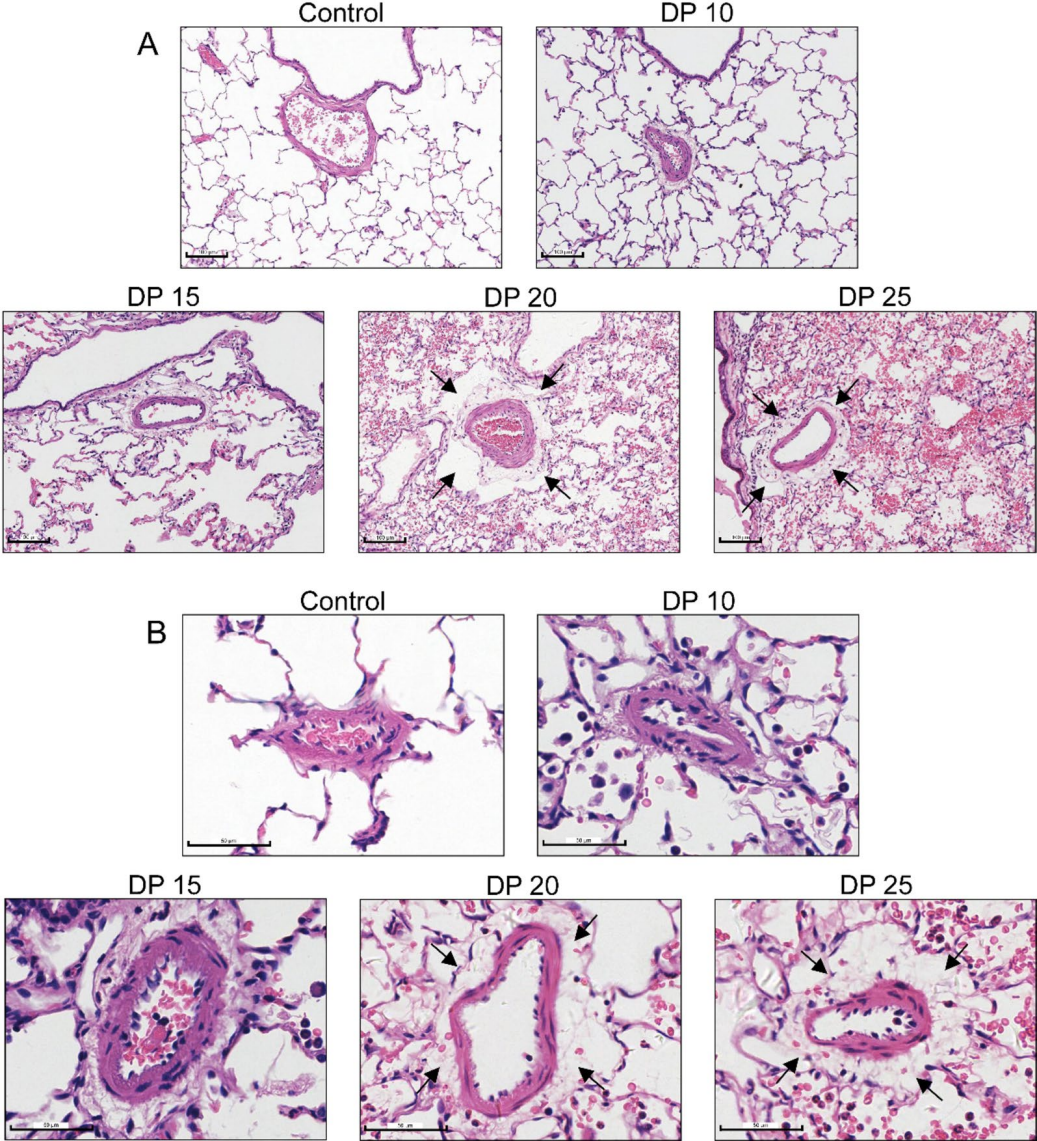
Histopathology of the pulmonary vessels. **A** Representative images of the hematoxylin and eosin staining of the pulmonary medium vessels. Magnification ×200. **B** Representative images of pulmonary small vessels. Magnification ×400. Pulmonary vessels of the 20 cmH_2_O and 25 cmH_2_O groups showed varying degrees of vessel wall thickening, extravascular edema, and inflammatory cell infiltration as indicated by black arrows

Then, we explored the effect of mechanical ventilation on lung histology in ALI. H&E staining showed that compared with the normal lungs of the control rabbits, the lungs of the ALI group demonstrated extensive lung injury, including interstitial edema, alveolar wall thickening, and inflammatory cell infiltration in the alveolar space. In addition, the lung injury tended to get worse as the driving pressure increased (Fig. 5A). Consistently, mechanical ventilation with ≥ 15 cmH_2_O driving pressure significantly increased the wet/dry weight ratio, lung injury scores, and BALF total proteins in a pressure-dependent manner (Fig. 5B–D). These data suggest that high driving pressure ventilation aggravates lung injury in ALI. We further compared the histopathology of the pulmonary vessels among different groups. Compared with the vessels of the control group, the pulmonary vessels of the ventilated ALI groups showed varying degrees of vessel wall thickening, extravascular edema, and inflammatory cell infiltration, which tended to aggravate with the driving pressure increasing (Fig. 6A). Similar phenomenon was observed in small pulmonary vessels (Fig. 6B). These data suggest that high driving pressure ventilation exacerbates pulmonary vessel injury in ALI.

### High driving pressure induces right heart remodeling in rabbits with ALI

**Fig. 7:**
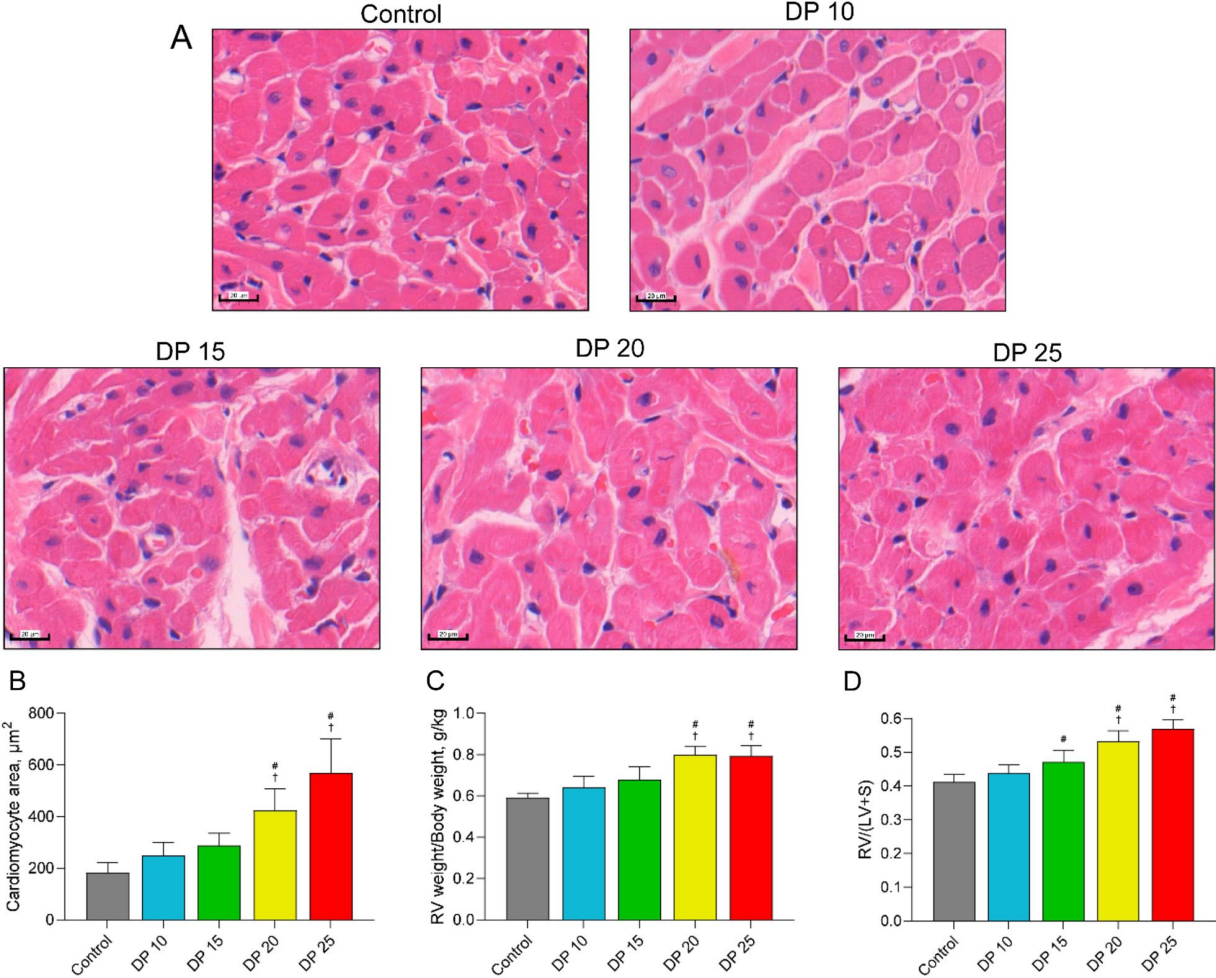
High driving pressure induced right ventricular hypertrophy in ALI. **A** Cardiac tissue sections were stained with hematoxylin and eosin. Images were acquired at magnification ×400. **B** Cross-sectional areas of myocytes. **C** Ratio of right ventricular weight/body weight. **D** Right ventricular/(left ventricular + septum) weight ratio. Data are presented as the means ± SD. ^#^*P* < 0.05 vs. control; *n* = 5. RV: right ventricular; RV/(LV + S): right ventricular/(left ventricular + septum) weight ratio

Next, we examined whether high driving pressure ventilation induces changes in the right ventricle in ALI. As expected, compared with control or low driving pressure, 20 cmH_2_O and 25 cmH_2_O ventilation significantly increased cross-sectional areas of cardiomyocytes, the ratio of right ventricular weight to body weight, and RV/[LV + S] (Fig. 7B–D, all *P* < 0.05). These results indicate an increase in right heart weight and suggest remodeling of the right ventricle in response to high driving pressure ventilation in ALI.

### High driving pressure ventilation induces pulmonary transcriptome alterations in rabbits with ALI

**Fig. 8:**
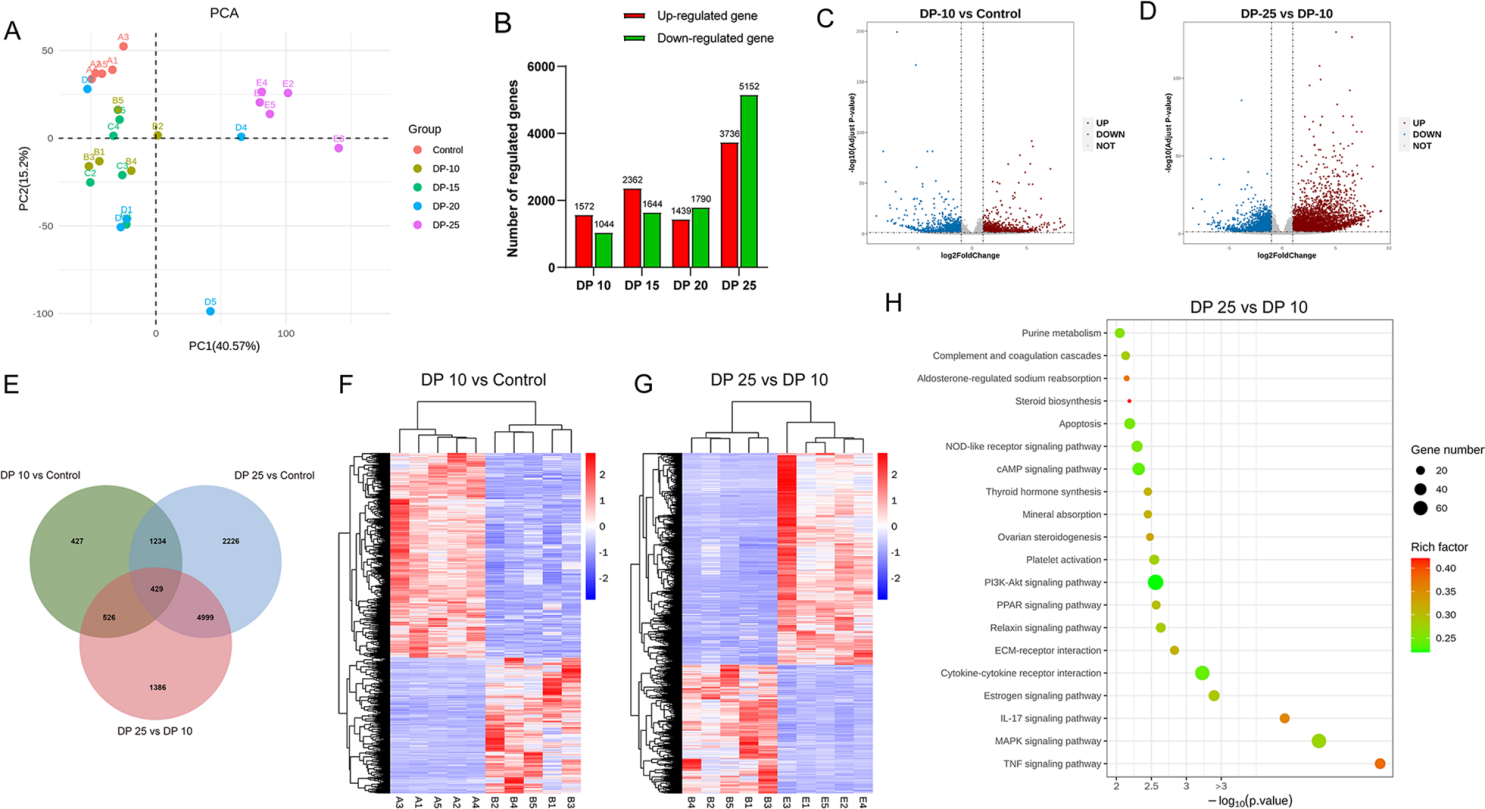
Transcriptomics analysis. **A** RNA sequencing was performed to identify differentially expressed genes in lung tissue samples from rabbits. Principal components analysis was conducted to evaluate genomic similarities of different groups. **B** Number of differentially expressed genes in each ventilation group vs. the control group. **C**, **D** Volcano plots of differentially expressed genes in 10 cmH_2_O group vs. control group (**C**) and 20 cmH_2_O group vs. 10 cmH_2_O group (**D**). **E** Venn diagram of the numbers of differentially expressed genes. **F**, **G** Heatmaps of differentially expressed genes in 10 cmH_2_O group vs. control group (**F**) and 20 cmH_2_O group vs. 10 cmH_2_O group (**G**). **H** Kyoto Encyclopedia of Genes and Genomes enrichment analysis of differentially expressed genes in 25 cmH_2_O group vs. 10 cmH_2_O group. DP: driving pressure; PCA: principal components analysis

To identify potential mechanisms of high driving pressure ventilation-induced pulmonary and cardiovascular abnormalities, we compared lung tissue transcriptomes among different groups. Principal components analysis revealed that the control cluster was separated from the ALI clusters, and the 25 cmH_2_O cluster was isolated from other ALI clusters (Fig. 8A), suggesting high driving pressure ventilation induces transcriptome alterations in ALI. The number of differentially expressed genes (DEGs) tended to increase as driving pressure raised (Fig. 8B). Specifically, a total of 2616 DEGs were identified in the 10 cmH_2_O group vs. the control group, including 1572 upregulated DEGs and 1044 downregulated DEGs (Fig. 8C). A total of 7340 DEGs were identified in the 25 cmH_2_O group vs. the 10 cmH_2_O group, including 2774 upregulated DEGs and 4566 downregulated DEGs (Fig. 8D). According to a Venn diagram, 10 mmH_2_O and 25 mmH_2_O groups shared 429 common DEGs (Fig. 8E). Heatmap demonstrated differential transcriptomes between 10 cmH_2_O and control groups (Fig. 8F) and between 25 cmH_2_O and 10 cmH_2_O groups (Fig. 8G). Kyoto Encyclopedia of Genes and Genomes pathway enrichment analysis revealed that the DEGs of the 25 cmH_2_O group vs. 10 cmH_2_O groups were mainly associated with the TNF, MAPK, IL-17, NOD-like receptor, and apoptosis signaling pathways (all *P* < 0.01; Fig. 8H).

### High driving pressure ventilation alters ferroptosis-related gene expression in the lung tissue of rabbits with ALI

**Fig. 9:**
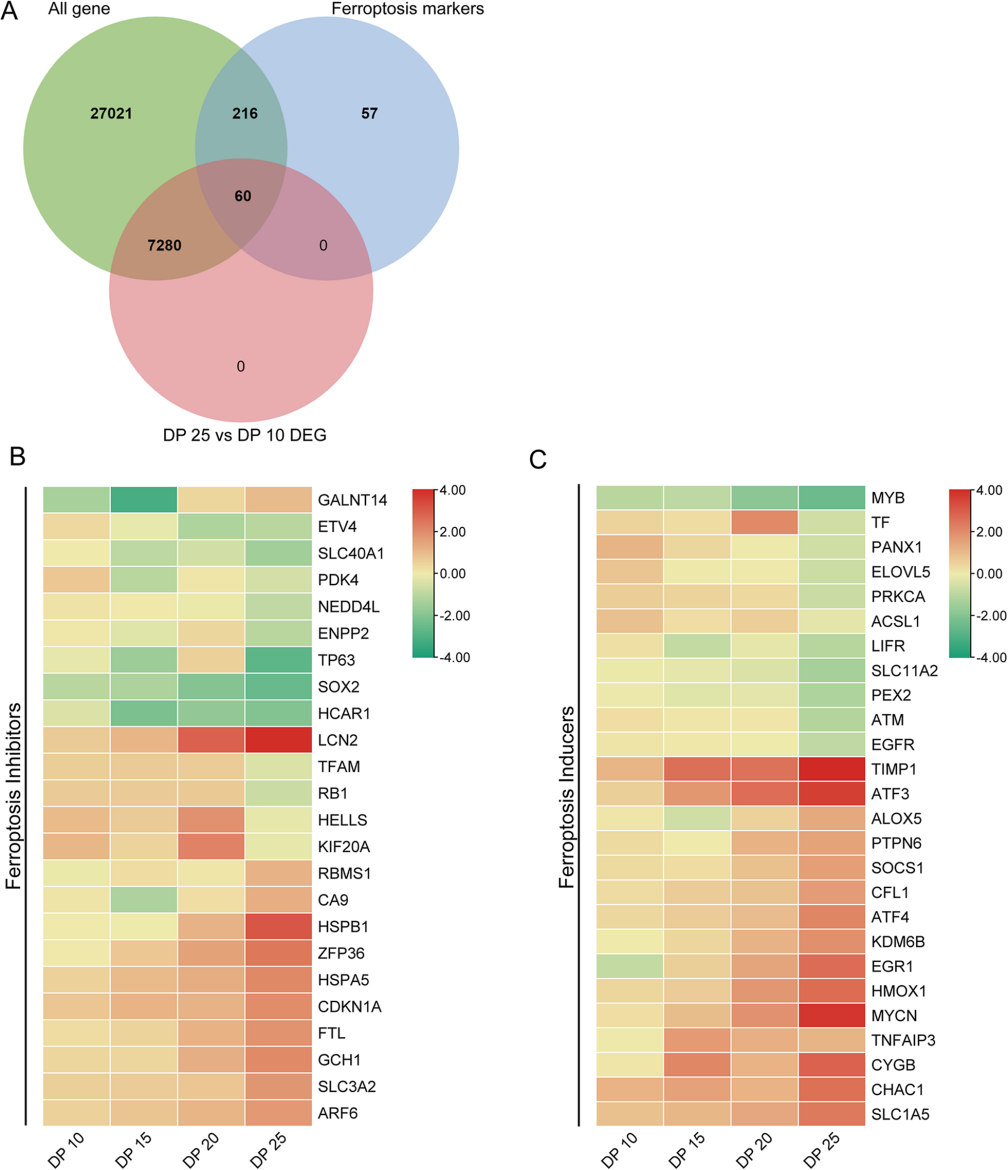
High driving ventilation induced alterations in ferroptosis-related gene in lung tissue of rabbits with ALI. **A** Venn diagram of the numbers of differentially expressed genes. **B** Heatmap of the expression of the genes related to ferroptosis suppression. **C** Heatmap of the expression of the genes related to ferroptosis induction

**Fig. 10:**
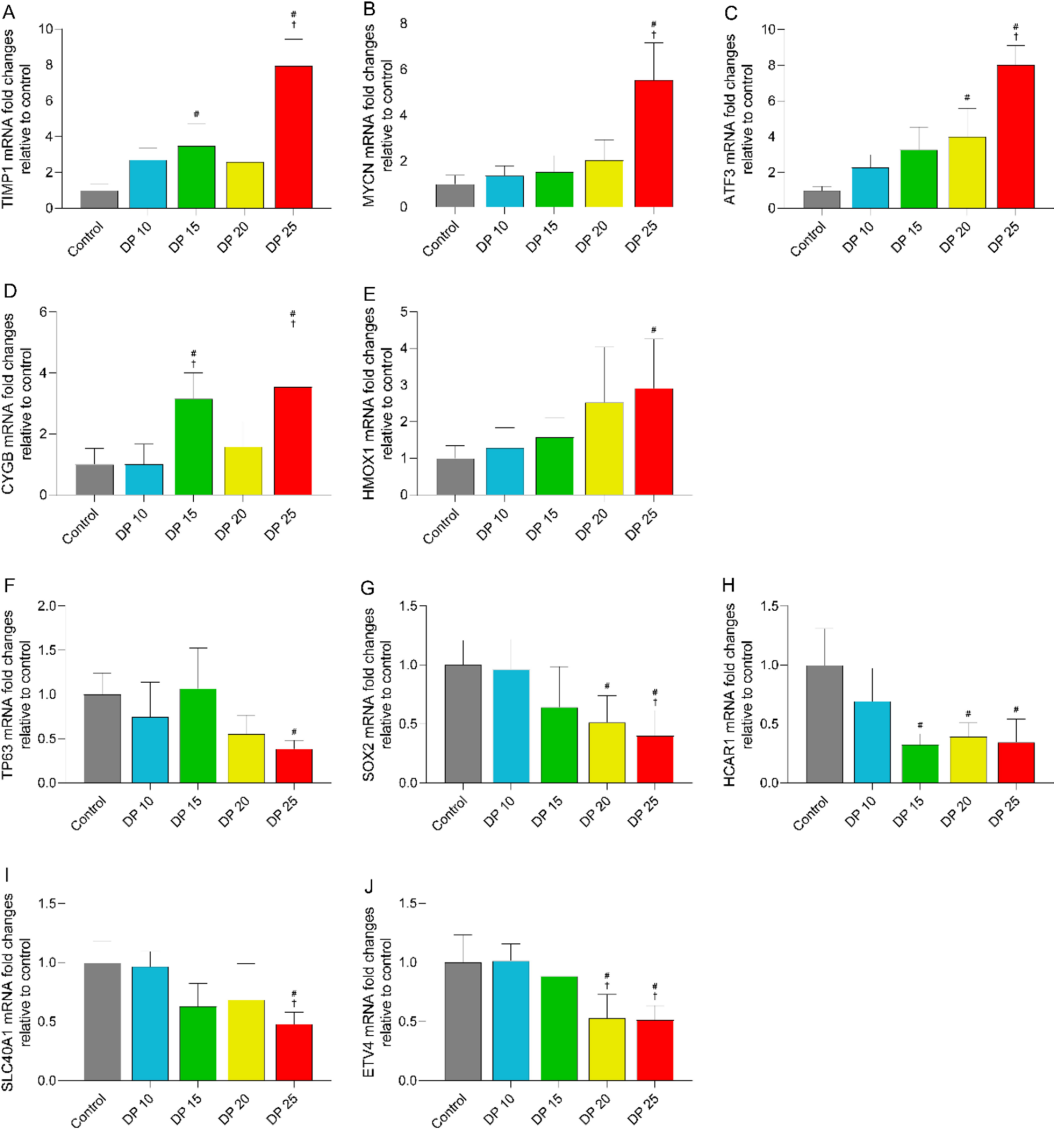
Validation of high driving ventilation-induced alterations in ferroptosis-related genes. Real-time PCR was performed to determine mRNA expression of the top five differentially expressed genes related to ferroptosis induction (**A**–**E**) and suppression (**F**–**J**) in lung tissue samples from rabbits. Data are expressed as the mean ± SD. ^#^*P* < 0.05 vs. control. ^†^*P* < 0.05 vs. 10 cmH_2_O; *n* = 5

Ferroptosis is a unique cell death mode driven by iron-dependent lipid peroxidation [13]. Recent studies have shown that ferroptosis is closely associated with the development of many lung disorders, including ALI/ARDS and pulmonary hypertension [14, 15]. Thus, we sought to identify ferroptosis-related DEGs associated with high driving pressure VIPH in ALI. We found 60 ferroptosis-related DEGs between 25 cmH_2_O and 10 cmH_2_O groups (Fig. 9A). The differential expression of the genes related to ferroptosis induction/suppression among different driving pressure groups are shown in Fig. 9B, C, respectively. It seems that as the driving pressure increased, the mRNA expression of the genes related to ferroptosis suppression tended to attenuate, whereas the mRNA expression of the genes related to ferroptosis induction became stronger. The results of qPCR confirmed the dysregulated expression of the top five genes related to ferroptosis induction/suppression in high driving pressure groups, especially in the 25 cmH_2_O group (Fig. 10A–J). These data suggest that enhanced ferroptosis may play an important role in high driving pressure ventilation-induced cardiopulmonary disorders in ALI.

## Discussion

To date, the animal model of VIPH in ALI has rarely been reported at least partially due to technical challenges and high mortality of experimental animals. In this study, we successfully established a VIPH model in rabbits with ALI through the right heart catheterization which determines the pulmonary artery pressure rapidly and easily. Our methods may provide useful information for other researchers. In this study, ALI was induced in rabbits by a two-hit injury combining acid aspiration and 1-h mechanical ventilation. The subsequent application of 7-h high driving pressure ventilation remarkably increased pulmonary arterial pressure and induced right ventricle hypertrophy in rabbits with ALI, confirming that mechanical ventilation led to acute pulmonary hypertension. The results of RNA sequencing of whole lung tissues suggested that enhanced ferroptosis might be associated with high driving pressure ventilation-induced cardiopulmonary disorders, providing some new information about the mechanism underlying the occurrence of ventilation-induced adverse events in ALI.

Patients with severe ALI/ARDS are frequently complicated by pulmonary hypertension, which is characterized by a progressive increase in pulmonary vascular resistance, leading to right ventricular dysfunction and ultimately right ventricular failure [16]. Pulmonary hypertension is clinically defined as mean pulmonary arterial pressure > 20 mmHg, measured by right heart catheterization [16]. Acute pulmonary hypertension in ARDS falls into group 3 pulmonary hypertension due to the presence of hypoxia and lung injury [17]. Pulmonary hypertension in ARDS patients was first described by Zapol et al. [18], with a prevalence of 20–25% during the first 3 days of protective mechanical ventilation [19–21]. We found that the PaO_2_/FiO_2_ value of the ALI group dramatically dropped from around 515.8 ± 27.3 mmHg at baseline to 144.5 ± 23.8 mmHg at T0. These results are consistent with a previous study which determined that driving pressure ≥ 18 cmH_2_O, PaCO_2_ ≥ 48 mmHg, and PaO_2_/FiO_2_ ratio < 150 mmHg that reflects the ventilatory strategy and lung injury severity are independent factors associated with right ventricular injury in ARDS patients [5]. The findings of this study suggest that mechanical ventilation with injurious parameters for a brief period of time may led to acute pulmonary hypertension and acute heart failure. These results are consistent with clinical findings of other studies [22, 23]. Thus, it could be concluded that the animal model developed in this study is appropriate for studying the impact of mechanical ventilation on acute pulmonary hypertension and acute failure in clinic. Driving pressure is used as a surrogate for tidal volume-related lung stress. Increased driving pressure exacerbates the detrimental effect of lung distension on pulmonary capillaries, thereby adversely affecting right ventricular afterload and function [24, 25]. In our study, high driving pressure ventilation aggravated pulmonary vascular damage and induced acute pulmonary hypertension and right heart hypertrophy in rabbits with ALI. A recent review suggests that cardiopulmonary protective ventilation strategies, such as limiting plateau and driving pressures, improving hypercapnia, and adjusting PEEP titration according to RV function, may improve outcomes in patients with RV failure [26]. However, this strategy needs to be evaluated in clinical practice. The animal model established in this study is ideal for preclinical studies.

In our study, we used RNA sequencing on whole lung tissues to explore the mechanisms underlying VIPH in ALI. We found differential expression of ferroptosis-related genes across various driving pressure groups. Ferroptosis is involved in lung injury across different cell types. Increased reactive oxygen species, epithelial cell apoptosis, and inflammation are important pathogenic mechanisms of ALI [27]. In immune cells, ferroptosis triggers immune responses by attracting antigen-presenting cells, such as macrophages, dendritic cells, and neutrophils to the injury site, thereby influencing disease progression [28]. In endothelial cells, ferroptosis can lead to increased vascular permeability, inflammation, and tissue damage, contributing to the progression and severity of lung injuries, such as ALI and ARDS [29]. In addition, endothelial cell ferroptosis mediates monocrotaline-induced pulmonary hypertension, highlighting the involvement of this cell death pathway in pulmonary vascular dysfunction [31]. These findings suggest that targeting ferroptosis in epithelial, immune, and endothelial cells could potentially serve as a therapeutic strategy for mitigating ALI and pulmonary hypertension.

Protective mechanical ventilation can generally improve the outcomes of patients with ARDS and pulmonary hypertension, but pulmonary vascular dysfunction is still present in 25% of the patients [21, 32]. ARDS is an inflammatory disease associated with endothelial cell injury and dysfunction [33] that may induce acute pulmonary vasoconstriction and subsequent pulmonary vascular remodeling. Thus, ARDS-related inflammation and ventilation-induced inflammation may be the central mechanism of the development of pulmonary hypertension in ARDS [34]. Consistent with previous studies, our study showed that the TNF, MAPK, IL-17, and NOD-like receptor signaling pathways were significantly enriched in high driving pressure ventilated lungs.

Ferroptosis is a type of nonapoptotic cell death that is dependent on iron and reactive oxygen species [35]. Ferroptosis contributes to the pathogenesis of lung diseases, including chronic obstructive pulmonary disease [36], asthma [29], pulmonary fibrosis [29], ischemia–reperfusion injury [37], ALI/ARDS [38, 39], and pulmonary hypertension [31]. In this study, the genes related to ferroptosis induction/suppression were differentially expressed in lung tissue exposed to high driving pressure ventilation, suggesting that ferroptosis is associated with VIPH in ALI. qPCR further confirmed the dysregulated expression of the top five genes related to ferroptosis induction/suppression exposed to high driving pressure up to 25 cmH_2_O. These results suggest that enhanced ferroptosis play a crucial role in high driving pressure ventilation-induced cardiopulmonary disorders in ALI. In ALI, ferroptosis inducers can exacerbate pulmonary edema and alveolar inflammation, along with high levels of cytokines (IL-1β, IL-6, and TNF-α), which can be reversed by ferroptosis inhibitors [40–42]. Iron overload, glutathione depletion, and downregulated ferritin expression have been observed in the lung tissue of intestinal ischemia–reperfusion-induced acute lung injury in mice [38]. Redox imbalance has been linked to ventilation [43]. Glutathione is commonly used as an indicator of cellular redox status. Pires et al. have reported that glutathione was significantly reduced in the ventilated lung compared with that in the lung with spontaneous breathing in mice [44]. Furthermore, cellular glutathione inhibits ferroptosis [45, 46]. Therefore, we speculate that mechanical ventilation might promote ferroptosis in lung tissue possibly by reducing glutathione, leading to lung injury and acute pulmonary hypertension. The mechanical ventilation-induced lung injury was further confirmed through histopathology observation. H&E staining revealed that the lungs of the ALI group had extensive damage, including interstitial edema, alveolar wall thickening, and inflammatory cell infiltration, and the injury became worse with increasing driving pressure. These results are consistent with a previous study, where mechanical ventilation altered the alveolar structure and thickened the alveolar space as well as a large number of inflammatory cells infiltrated in lung tissue [47]. Other studies have shown that mechanical ventilation induces macrophage activation, recruitment of inflammatory cells to the lungs, amplifies the inflammatory response [48, 49], and affects the extracellular matrix [50]. Therefore, we will investigate the effect of short period mechanical ventilation on ferroptosis, inflammation, and composition of extracellular matrix of different cell types, such as alveolar epithelial cells and endothelial cells, in lung tissues, in our future studies. Furthermore, mechanical ventilation led to pressure-dependent increase in the wet/dry weight ratio, lung injury scores, and BALF total proteins, suggesting that driving pressure ventilation exacerbates lung injury in ALI. Overall, these findings suggest that high driving pressure ventilation causes acute pulmonary hypertension.

In our study, the use of the rabbit model allowed for more accurate assessment of respiratory mechanics compared to smaller animal models. Our findings indicate that high driving pressure ventilation can lead to acute pulmonary hypertension and right ventricular enlargement. Therefore, it is important to not only focus on lung protection during mechanical ventilation but also prioritize right heart protection [51]. For patients undergoing high driving pressure ventilation, particularly those with ARDS and acute cor pulmonale, adjusting ventilation strategies is crucial. If ventilation parameters cannot be sufficiently reduced, timely consideration of extracorporeal life support is warranted for optimal support and management.

Our model utilized hydrochloric acid-induced lung injury, which, clinically, constitutes a relatively small proportion of ALI cases compared to those caused by infection. In addition, the etiology of ALI in clinical settings is multifactorial, with significant heterogeneity among individuals. As a result, our model may not fully replicate the complexity of clinical ALI. However, the hydrochloric acid-induced lung injury model accelerates lung damage and oxygenation impairment, allowing for a rapid simulation of severe ALI observed in clinical scenarios. These limitations should be taken into consideration when interpreting the findings and extrapolating them to human ALI/ARDS. Future research should explore additional models and incorporate a broader range of ALI etiologies to enhance the clinical relevance of the findings.

## Conclusions

In summary, we established a rabbit model of VIPH in ALI. High driving pressure ventilation aggravated pulmonary vascular damage while inducing acute pulmonary hypertension and right heart hypertrophy. In addition, we identified differentially expressed ferroptosis-related genes in high driving pressure ventilated lungs. Our findings suggest a potential association between ferroptosis and the development of VIPH in ALI, warranting further investigation to fully understand this relationship.

## Data Availability

All data generated or analysed during this study are included in this published article.

## Abbreviations

ARDS: Acute respiratory distress syndrome
VIPH: Ventilation-induced pulmonary hypertension
DEGs: Differentially expressed genes
FiO_2_: Fraction of inspired oxygen
PEEP: Positive end-expiratory pressure
MAP: Mean arterial pressure
mPAP: Mean pulmonary artery pressure
RV: Right ventricle
PaCO_2_: Pressure of arterial carbon dioxide
